# Inherited unbalanced translocation (4p16.3p15.32 duplication/8p23.3p23.2deletion) in the four generation pedigree with intellectual disability/developmental delay

**DOI:** 10.1186/s13039-021-00552-3

**Published:** 2021-07-08

**Authors:** Dongmei Hao, Yajuan Li, Lisha Chen, Xiliang Wang, Mengxing Wang, Yuexin Yu

**Affiliations:** 1 Department of Reproductive Medicine Center, General Hospital of Northern Theater Command, Shenyang, Liaoning 110016 People’s Republic of China; 2 General Hospital of Northern Theater Command, Postgraduate Training Base of Jinzhou Medical University, No. 83. Wenhua Road, Shenhe District, Shenyang, Liaoning 110016 People’s Republic of China

**Keywords:** 4p duplication, 8p deletion, Intellectual disability, Inherited, Single-nucleotide polymorphism

## Abstract

Chromosomal copy number variants (CNVs) are an important cause of congenital malformations and mental retardation. This study reported a large Chinese pedigree (4-generation, 76 members) with mental retardation caused by chromosome microduplication/microdeletion. There were 10 affected individuals with intellectual disability (ID), developmental delay (DD), and language delay phenotypes. SNP array analysis was performed in the proband and eight patients and found all of them had a microduplication of chromosome 4p16.3p15.2 and a microdeletion of chromosome 8p23.3p23.2. The high-resolution karyotyping analysis of the proband had unbalanced karyotype [46, XY, der(8)t(4;8)(p15.2;p23.1)mat], his mother had balanced karyotype [46, XX, t(4;8) (p15.2;p23.1)], whereas his father had normal karyotype [46,XY]. Fluorescence in situ hybridization (FISH) analysis further confirmed that the proband’s mother had a balanced translocation between the short arm terminal segment of chromosome 4 and the short arm end segment of chromosome 8, ish t(4;8)(8p + ,4q + ;4p + ,8q +). In conclusion, all the patients inherited chromosomes 8 with 4p16.3p15.2 duplication and 8p23.3p23.2 deletion from their parental balanced translocation, which might be the cause of the prevalence of intellectual disability. Meanwhile, 8p23.3p23.2 deletion, rather than 4p16.3p15.2 duplication might cause a more severe clinical syndrome.

## Introduction

Intellectual disability (ID), also referred as cognitive impairment or mental retardation, is characterized by a substantially below-average score on tests of mental ability or intelligence (intellectual quotient (IQ) < 70), and limitations in adaptive behaviors. These abnormal phenotypes normally show up before the age of 18 years and 2 months [1]. It is estimated that ID affects approximately 1% to 3% of the general population [2]. The etiology of ID is complex and can be the result of genetic and/or environmental factors. Clinically, it is a broad diagnosis encompassing a wide variety of phenotypes and severities from simple ID to complex dysmorphia, epilepsia, autism spectrum disorder (ASD). Approximately 15% to 40% of ID is due to genetic factors [2]. Chromosomal copy number variations (CNVs) are known to be an important component of genetic variants and play an important role in the etiology of ID [3].

Here, we reported an inherited ID / DD (developmental delay) case with chromosome unbalanced translocation between 4p and 8p using SNP array in a large 4-generation Chinese family (76 members). All the patients inherited the same derived chromosome 8 with 4p16.3p15.2 duplication and 8p23.3p23.2 deletion from their parental balanced translocation. Previous studies have found that chromosome 4p terminal duplication was associated with physical overgrowth, heavy facial features, and mild to moderate mental handicap [4, 5], and chromosome 8p deletion was associated with 8p deletion syndrome including DD, ID, congenital heart defects (CHD), genital abnormality, neuromental disorder, etc. [6, 7]. Some patients had extra facial dysmorphic features including microcephaly, low-set ears, depressed nasal bridge, serrated teeth, etc. To the best of our knowledge, this is a report involving the largest pedigree that is due to a duplication 4p16.3p15.32 and a deletion 8p23.3p23.2.

## Patients and methods

### Proband and family history investigation

**Fig. 1:**
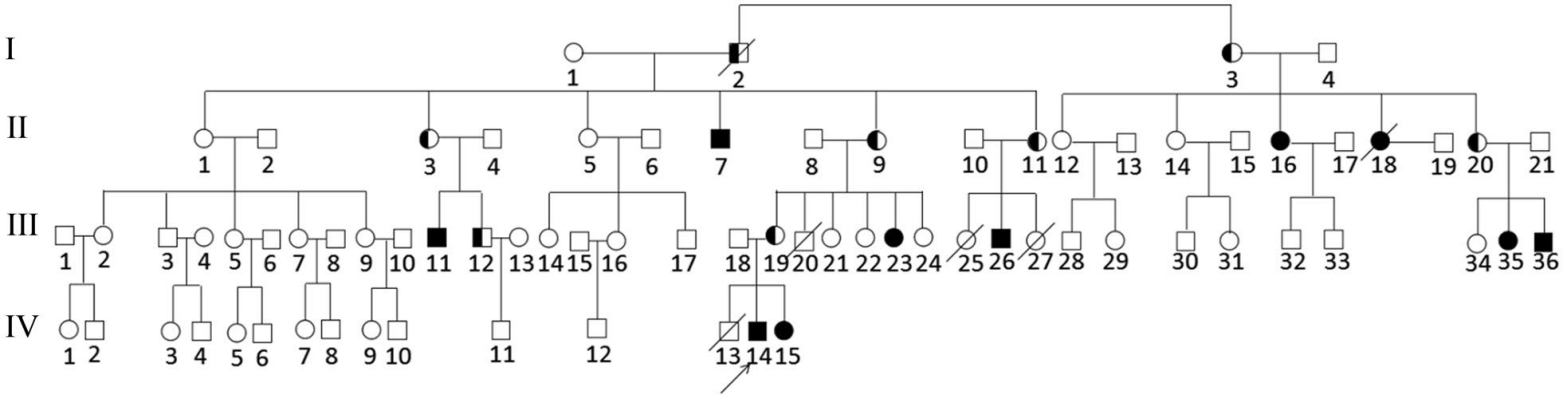
The pedigree of a large Chinese family with development disability and intellectual disability. The proband is indicated with an arrow. The circle presents female and the square presents male. The circle or square with white means normal individual, the black means patients, and the carrier is shown as half white and half black

A pedigree diagram of the patient is shown in Fig. 1. The proband (IV-14) was a 4-year-old boy. He was born by vaginal delivery at 36 weeks of gestation to the nonconsanguineous and healthy parents. According to WHO Child Growth Standards, his birth weight was at the 1st percentile (2.2 kg), birth length was at the 5th percentile (47 cm), and head circumference was at the 3rd percentile (32 cm) [8]. There were no facial dysmorphic features and other malformations. He had been in the incubator of a local hospital for about one month because of unexplained premature delivery and low birth weight. Postnatal development indicators showed that he had DD. He sat alone at 10 months, walked at 20 months, and tiptoed slightly. He always fell over because of imbalance. Additionally, language disability was obvious. Till now, the proband can only say three words, which are pronounced through mouth and nasal cavity. It was very difficult to communicate with and understand him. He had no feeding difficulties. At age of 4 years and 2 months, the IQ test displayed that he had intellectual disability (IQ 55) and achieved an Age Equivalent (AE) of 27.5 months. The test of Social Living Ability of Infant-Junior Middle School Students was measured. The total score was 22 (standard score 7), suggesting that he had a medium problem in his social living ability. On the other hand, his height was at the 85th percentile (110 cm) [8] and his physical development was normal and comparable with other healthy boys of the same age. Brain magnetic resonance imaging (MRI) did not reveal any anomalies. Currently, he was receiving preschool education in a kindergarten, but it was difficult for him to learn from the teacher. His hands always kept playing with the surrounding interesting objects. When he heard forbidden instructions, he showed impulsive and aggressive behavior.

The family history investigation showed that there were 9 other affected individuals aged between 2 and 51 years in this Chinese pedigree. All of them had the same phenotypes as the proband except that one of them (II-18) had seizure in addition to ID/DD. We cannot have more information about her because she had died of an accident. While the patients were noted with ID/DD, they neither received any therapy from medical services, nor accepted any education from a special school. At present, these adult patients (II-7, II-16 and III-11) can take care of themselves, labor on the farmlands with their family members and communicate orally. Because they had not received vocal training, the pronunciation was inarticulate. Although they had made slight improvement in language communication ability, they had many difficulties in understanding complex or logic expressions. When facing unfamiliar persons or things, these adult patients could be attracted, but they would keep silent and gaze at them curiously. In this family, the children (III-20, III-27) were diagnosed with the congenital heart defects (CHD) and development delay, and unfortunately died at the age of 4 and 2 years, respectively. The infant patients (III-25) and (IV-13) were diagnosed with hydrocephalus, and hydrocephalus and cleft spine, respectively. And both of them died after birth. We selected the patients (II-7, II-16, III-11, III-23, III-26, III-35, III-36, IV-14, IV-15) and the proband’s parents (III-18, III-19) as research subjects. The history of pregnancy and miscarriages of all family members was collected. This study was approved by the Ethics Committee of General Hospital of Northern Theater Command, and written informed consent was obtained from the patients’ parents. Written informed consent was also obtained from the patients’ parents for publication of this case.

## Methods

### SNP array analysis

II-18 did not have a blood sample because of death, so totally 9 samples were tested. Genomic DNA from the patients (II-7, II-16, III-11, III-23, III-26, III-35, III-36, IV-14, IV-15) and the proband’s parents (III-18, III-19) was extracted from 2 ml of peripheral blood in EDTA using the QIAamp DNA Mini Kit (Qiagen, Hilden, Germany). Single nucleotide polymorphism (SNP) array analysis was performed using Infinium Global Screening Array (Illumina, San Diego, CA) containing about 700 000 markers for genome-wide tag SNPs and other regions of known cytogenetic importance. Automated detection of copy number changes was carried out using the cnvPartition algorithm (versions 1.2.1 to 3.1.6) in KaryoStudio software (Illumina). We evaluated the CNVs with the information provided by the Online Mendelian Inheritance in Man database (OMIM) [9], the in Humans using Ensembl Resources Database (DECIPHER) [10], the Database of Genomic Variants (DGV) [11], CHOP database [12] and literature.

### Cytogenetic analysis

Chromosome karyotyping analysis was detected on high-resolution G-banded metaphases prepared from the freshly drawn blood of proband (IV-14) and his parents (III-18, III-19) using the laboratory’s standard procedures. Twenty metaphases were analyzed for the sample according to the International System for Human Cytogenetic Nomenclature (ISCN 2016) [13].

### *Fluorescence *in situ* hybridization analysis*

This analysis was done by Be Creative Lab(Beijing) Co. Ltd, using whole chromosome painting probes for chromosome 4pter/4qter and 8pter/8qter. Fluorescence in situ hybridization (FISH) was performed on metaphase slides for the proband’s mother according to the standard method.

## Results

**Fig. 2:**
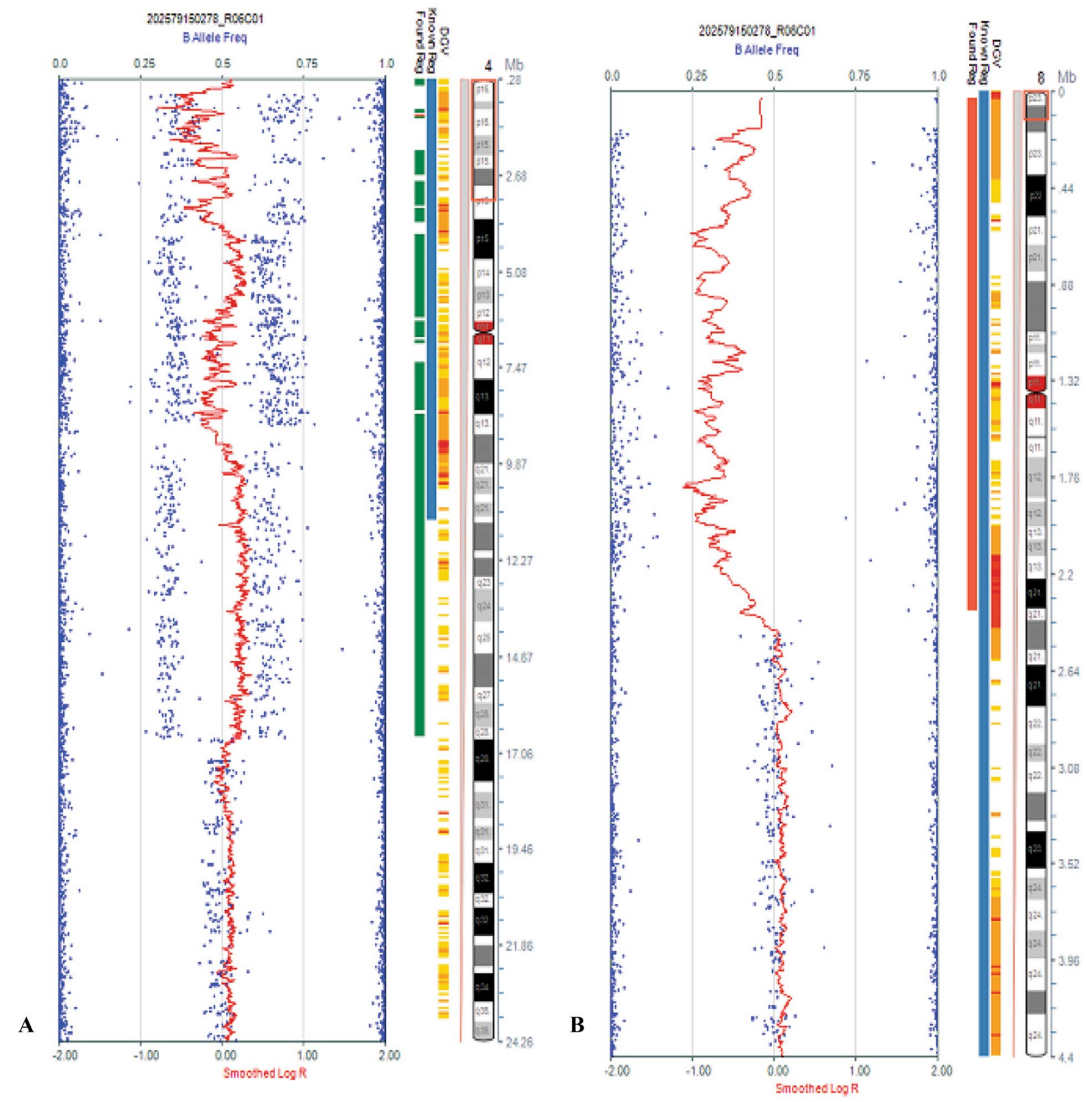
Single-nucleotide polymorphism (SNP) array identified a duplication 4p16.3p15.32 **(a)** and a deletion 8p23.3p23.2 **(b)** in the patients

SNP array showed that the proband (IV-14) and other patients had a 16.706 Mb duplication of 4p16.3p15.32 and a 2.33 Mb deletion of 8p23.3p23.2. Molecular karyotype: arr [hg19] 4p16.3p15.32 (35030–16705689) × 3, 8p23.3p23.2 (33142–2372867) × 1 (Fig. 2). The high-resolution karyotyping analysis showed that proband had unbalanced karyotype [46,XY,der(8)t(4;8)(p15.2;p23.1)mat], his mother had balanced karyotype [46,XX, t (4;8) (p15.2;p23.1)], while his father had normal karyotype [46,XY]. FISH analysis further confirmed that the proband’s mother had a balanced translocation between the short arm terminal segment of chromosome 4 and the short arm terminal segment of chromosome 8, ish t(4;8)(8p + ,4q + ;4p + ,8q +) [13]. So, we finally detected that the chromosome aberration of the proband and other patients detected through SNP array were inherited from derived chromosome 8 [der(8)t(4;8)(p15.2;p23.1)] and normal chromosome 4 of balanced translocation carrier [t(4;8)(p15.2;p23.1)] in this intellectual family pedigree.

## Discussion

We report an unbalanced translocation involving chromosomes 4p and 8p in ten affected family members of a 4-generation pedigree, resulting in clinical manifestations of intellectual disability, growth retardation and language delay. Using SNP array and cytogenetic analysis, the tested nine patients were identified to carry a derived chromosome 8 [der(8)t(4;8)(p15.2;p23.1)] with a duplication of 4p16.3p15.32 and a deletion of 8p23.3p23.2. Ten patients were born by their healthy parent with a balanced translocation of chromosomes 4 and 8 [46,XX,t(4;8)(p15.2;p23.1)]. The derived chromosome 8 has been inherited for four generations in this family pedigree. We speculate that the individuals (III-20, III-25, III-27, IV-13) who had the serious congenital malformation and had been dead may inherit other kinds of lethally unbalanced gamete from balanced translocation carrier in this family.

It was reported that chromosome 4p terminal pure duplication was associated with physical overgrowth [4]. Furthermore, the dosage effect of fibroblast growth factor receptor gene 3 (FGFR3), which is located at 4p16.3, was suggested to relate to abnormal growth [5]. That is, a single dose leads to growth failure and a triple dose to physical overgrowth. Meanwhile, they also found that the partial monosomy or trisomy of the other chromosome involved in each of the translocations did not affect physical overgrowth or growth failure phenotype. Until now, there were more than 60 reports involving 4p16.3p15.32 pure duplication in the Database of Chromosomal Imbalance and Phenotype in DECIPHER [9]. Among that, # 392,041 had a 16.9 Mb duplication region similar to our study. It was characterized with ID, hiatus hernia, feeding difficulties in infancy, attached earlobe, proportionate short stature, cubitus valgus, small hand, and muscular hypotonia. However, the phenotype associated with overgrowth syndrome was not found in our study.

The 8p deletion syndrome is most commonly associated with major congenital anomalies, such as congenital heart defects (CHD) and congenital diaphragmatic hernia (CDH). It was reported that haplo-insufficiency of the genes (*GATA4*, *SOX7 or NEIL2*) involving chromosome 8p23.1 region might lead to CDH or CHD [14, 15]. In our study, no congenital malformations were found int the patients with 8p23.1 deficiency. Table 1 summarized the clinical characteristics of the patients with 8p23.2 → pter pure deletion [16–19] and compared them. In this table, the most common features including DD, ID, behavioral problems and balance/coordination problems, without microcephaly, facial dysmorphism, autism spectrum disorder, epilepsia, genital abnormality. The size of the deletion region ranged from 2.06 to 7.02 Mb, and the deletion region detected in our study was 2.373 Mb. A 12-year-old male with a missing fragment of approximately 2.4 Mb at 8p23.2 had autistic disorder, epilepsy, and behavioral problems [17]. Therefore, we found this deletion region was associated with ASD. There were about 32 records in the Database of DECIPHER for 8p23.3p23.2 alone deletion. They had clinical differences among these patients. In addition to ID/DD, these individuals had more serious phenotypes.

**Table 1:** Contrast of the clinical features of patients with 8p23.3p23.2 pure deletion in the present study

Patient	Wu et al	Chien et al	Burnside et al			Shi et al	Present study
			No.1	No.2	No.3		
Age/Sex	1**y**/F	12**y**/M	2**y**/F	4**y**/M	26**y**/M	5**y**/M	4**y**/M
Size(Mb)	2.06	2.4	3.6	4.8	7.02	6	2.373
Microcephaly	+	−	+	+	−	+	−
Facial dysmorphism	+	−	+	+	+	+	−
Epilepsia	−	+	−	−	−	−	−
Balance/co-ordination problems	−	−	+	+	−	+	+
Autism spectrum disorder	N.D	+	−	−	−	+	−
Behavioural problems (type)	N.D	ADHD, impulsivity	−	−	Psychology, neurology	ADHD, hyperactivity, impulsivity	Hyperactivity, impulsivity
Candidate gene	*DLGAP2*, *CLN8*, *ARHGEF10*	*DLGAP2*, *CLN8*, *ARHGEF10*	*DLGAP2*, *CLN8*, *ARHGEF10*, *CSMD1*			*DLGAP2*, *CLN8*, *ARHGEF10*,*CSMD1*	*DLGAP2*, *CLN8*, *ARHGEF10*

*F* female, *M* male, *y* year**, **− feature absent, + feature present, *N.D*. not descriped, *ADHD* attention-deficit hyperactivity disorder

Few cases of unbalanced translocation with both chromosome 4 short arm terminal duplication and chromosome 8 short arm terminal deletion have been reported. The recently published literature is summarized in Table 2 [20–22]. A 5-month-old baby with facial deformity, heart defect, and abnormal genitourinary system was too young to exhibit developmental abnormalities [20]. Other patients reported having more severe phenotypes in this study [21]. For example, a 21-year-old male patient had severe neurological developmental disorders. He carried a de novo unbalanced translocation [der(8)t(4;8)(p16.1 → pter; 23.1 → pter)] detected by SNP microarray. Two siblings were identified as having ID and ASD [22]. In our study, no dysmorphism were detected in the patient except for DD/ID, and there were not severe clinical symptoms, such as ASD, repetitive behavior or obsessive compulsive disorder.

**Table 2:** Contrast of the clinical features of patients with 4p duplication and 8p deletion with our patient

Patients	Škrlec et al	Sagar et al	Reis et al		Our patient
			No.1	No.2	
Age/Sex	5 m/F	25y/F	11y/M	8y/F	4y/F
Karyotype	der(8)t(4;8) (p16.1;p23.1)	der(8)t(4;8) (p16.1 → pter; p23.1 → pter)	der(8)t(4;8)(p16.2;p23.3)	der(8)t(4;8) (p16.2;p23.3)	der(8)t(4;8)(p15.2;p23.1)
DD	N.D	N.D	+	+	+
ID	N.D	N.D	+	+	+
ADHD	N.D	+	−	−	−
Facial abnomalies	+	+	−	−	−
Language delay	N.D	+	+	+	+
Autism	N.D	+	+	+	−
Cardiovascular anomalies	atrial septal defect	+	−	−	−
Unstable emotion	N.D	+	+	+	+
Behavioural problems (type)	N.D	Repetitive behavior	Aggressive behavior	−	−
Hyperactivity	N.D	+	−	+	+
Overgrowth	N.D	Macrocephalic	−	−	−
Learning disability	N.D	+	+	+	+
Abnormal walking posture	N.D	+	−	−	+
Skeletal anomalies	Clinodactyly, widely spaced nipples, third toe low inserted	a mild thoracic scoliosis concave	−	−	−
Other	−	Hypertrichosis of the eyelashes;	−	−	−

*m* month, *y* year, *F* female, *M* male, − feature absent, + feature present, *N.D*. not descriped, *DD* developmental delay, *ID* intellectual disability, *ADHD* attention-deficit hyperactivity disorder

Comparing cases from literature and database, we concluded that deletion of 8p23.3p23.2, rather than duplication of 4p16.3p15.2, causes a more severe clinical syndrome. The 8p23.3P23.2 deletion region contains 6 OMIM genes, including *FBXO25*, *DLGAP2*, *CLN8*, *ARHGEF10*, *ERICH1-AS1*, and *MYOM2*. Currently, the *DLGAP2*, *CLN8*, *ARHGEF10* genes on the 8p23.3 chromosome region are known to be expressed in the brain, and are considered as candidate genes for neurological diseases (Table 1). The autosomal recessive mutations of *CLN8* gene were related to two abnormal phenotypes: progressive epilepsy and mental retardation (EPMR), also known as Northern epilepsy syndrome [23] and a variant late-infantile neuronal ceroid lipofuscinoses (v-LINCL), characterized by earlier onset, faster progression of the disease with speech delayed, developmental delay and seizures [24, 25]. The Late-Infantile-Onset Neuronal Ceroid Lipofuscinosis (LINCL) was related to homozygous mutation of CLN8 gene [26]. In the early stage of illness, the patient walked late, had an unstable gait, tiptoe and falled frequently, and had a delayed language development. And one patient with v-LINCL inherited a de novo CLN8 heterozygous mutation in the paternal line and a de novo 8p23.3 deletion in the maternal line [15]. According to the above literature, the early clinical symptoms of the proband witness and his sister in this study may be related to the heterozygous deletion of CLN8 gene. The *DLGAP2* gene has 12 exons encodes the discs, large (Drosophila) homolog-associated protein 2, and is expressed in the brain, testis, kidney and thyroid [27]. DLGAP2 protein has been connected to a diversity of neurological disorders including schizophrenia [28] fragile x mental retardation [29], post traumatic stress disorder [30], ASD [31, 32]. The *ARHGEF10* gene contains 22 exons, and encodes a guanine nucleotide exchange factor (GEF), which belongs to the Rho family of GTPase proteins (RhoGEFs) with Dbl homology (DH) domain [33]. The *Arhgef10*-depleted mice showed mental and behavior disorders [34]. This revealed that *ARHGEF10* gene contributes to neural morphogenesis and connectivity. Some studies have claimed that *ARHGEF10* was also associated with schizophrenia [35]. In the present study, since the nervous system symptoms are the main clinical features, we suggested that *CLN8*, *DLGAP2* and *ARHGEF10,* as candidate genes in the 8p23.3p23.2 deletion region, might explain the presented abnormalities for patients.

In conclusion, using SNP array and FISH, we detected a derived chromosome 8 with a duplication 4p16.3p15.32 and a deletion 8p23.3p23.2, which had been passed on for four generations in a large Chinese family. Moreover, the duplication of 4p16.3p15.2 and deletion of 8p23.3p23.2 might be the cause of the ID. Besides, *DLGAP2*, *CLN8* and *ARHGEF10* can be good candidate genes to in part explain the intellectual and developmental abnormalities for the patients.

## Data Availability

Data are available upon request.
